# Cutaneous Sensitivity Across Regions of the Foot Sole and Dorsum are Influenced by Foot Posture

**DOI:** 10.3389/fbioe.2021.744307

**Published:** 2022-01-13

**Authors:** Simone G.V.S. Smith, Maiya K. Yokich, Shawn M. Beaudette, Stephen H. M. Brown, Leah R. Bent

**Affiliations:** ^1^ Department of Human Health and Nutritional Science, University of Guelph, Guelph, ON, Canada; ^2^ Department of Kinesiology, Brock University, St. Catharines, ON, Canada

**Keywords:** cutaneous mechanoreceptors, posture, foot skin, mechanical deformation, skin stretch, tactile sensitivity, spatial acuity, biofeedback

## Abstract

Understanding the processing of tactile information is crucial for the development of biofeedback interventions that target cutaneous mechanoreceptors. Mechanics of the skin have been shown to influence cutaneous tactile sensitivity. It has been established that foot skin mechanics are altered due to foot posture, but whether these changes affect cutaneous sensitivity are unknown. The purpose of this study was to investigate the potential effect of posture-mediated skin deformation about the ankle joint on perceptual measures of foot skin sensitivity. Participants (*N* = 20) underwent perceptual skin sensitivity testing on either the foot sole (*N* = 10) or dorsum (*N* = 10) with the foot positioned in maximal dorsiflexion/toe extension, maximal plantarflexion/toe flexion, and a neutral foot posture. Perceptual tests included touch sensitivity, stretch sensitivity, and spatial acuity. Regional differences in touch sensitivity were found across the foot sole (*p* < 0.001) and dorsum (*p* < 0.001). Touch sensitivity also significantly increased in postures where the skin was compressed (*p* = 0.001). Regional differences in spatial acuity were found on the foot sole (*p* = 0.002) but not dorsum (*p* = 0.666). Spatial acuity was not significantly altered by posture across the foot sole and dorsum, other than an increase in sensitivity at the medial arch in the dorsiflexion posture (*p* = 0.006). Posture*site interactions were found for stretch sensitivity on the foot sole and dorsum in both the transverse and longitudinal directions (*p* < 0.005). Stretch sensitivity increased in postures where the skin was pre-stretched on both the foot sole and dorsum. Changes in sensitivity across locations and postures were believed to occur due to concurrent changes in skin mechanics, such as skin hardness and thickness, which follows our previous findings. Future cutaneous biofeedback interventions should be applied with an awareness of these changes in skin sensitivity, to maximize their effectiveness for foot sole and dorsum input.

## Introduction

Therapeutic and ergogenic interventions have recently focused on the use of biofeedback to improve function and performance. Understanding of skin function is essential for development of biofeedback interventions such as athletic taping, haptics, bracing, prosthetics, and clothing design. The sensation of touch is transduced by four classes of cutaneous mechanoreceptors that provide tactile feedback and respond to mechanical deformation of the skin (Johansson and Vallbo, 1979; Macefield, 2005; Strzalkowski et al., 2017). Receptors are classified by receptive field size, with small defined boundaries categorized as Type 1 and large undefined boundaries as Type 2. They are further classified by afferent firing, as fast adapting (FA) or slow adapting (SA) based on response to indentation. Each group of mechanoreceptors responds differently to stimuli such as vibration, stretch, stroking, and sustained indentation based on their properties, which are due to their morphology and location of the receptor endings in the skin tissue matrix (Johnson, 2001; Macefield, 2005).

Skin on the foot sole and dorsum is of particular interest for sensory augmentation as it plays a crucial role in the control of gait (Eils et al., 2004; Perry et al., 2001; Howe et al., 2015; 2018), postural stability (Stål et al., 2003; Meyer et al., 2004; Hageman et al., 1995), and balance control (Kavounoudias et al., 1998). Interventions to enhance information from foot mechanoreceptors have recently been implemented to improve sensory input in athletes, older adults, and patients with neurophysiological deficits (Novak and Novak, 2006; Lipsitz et al., 2015; Miranda et al., 2016). Vibratory insoles, in particular, have shown promising results, improving measures of balance and gait in older adults (Lipsitz et al., 2015), as well as in clinical populations such as those with diabetes mellitus, Parkinson’s disease, or post-stroke patients (Novak and Novak, 2006). Vibrating insoles have also shown promise in improving athletic performance through increased speed during agility tasks (Miranda et al., 2016).

The sensitivity of cutaneous mechanoreceptors on the foot sole are differentially affected based on the mechanical properties of the surrounding skin tissues (Strzalkowski et al., 2015b), and mechanics and sensitivity of the skin on the back have been shown to be influenced by spine posture (Beaudette et al., 2017a; Beaudette et al., 2017b). Specifically, Beaudette and colleagues (2017a, 2017b) showed altered trunk dorsum mechanics in response to changing posture, such as trunk flexion and extension, where movements into flexion resulted in harder, thinner skin on the back, whereas extension caused the skin to retract, becoming softer and thicker (Beaudette et al., 2017a). Changes to skin mechanics on the trunk were correlated to modifications in perceptual skin sensitivity. Skin compression, which occurred with trunk extension, increased sensitivity during tactile testing which targeted the FAI/II and SAII mechanoreceptors but sensitivity was shown to decrease when testing SAIs. The opposite response occurred with skin stretch. In the foot sole, Strzalkowski and colleagues (2015b) reported in a neutral foot position, harder and thicker skin was less sensitive to dynamic tactile stimuli compared to thinner and softer regions. Our lab has recently demonstrated that skin mechanics of the foot sole and dorsum, including stretch, thickness, and hardness, can be altered with ankle posture (Smith et al., 2019). Therefore, the purpose of the current study was to investigate the potential effect of posture-mediated skin deformation about the ankle joint on perceptual measures of foot skin sensitivity. As skin mechanics are altered with ankle posture, it can be hypothesized that such postures could lead to modifications in foot skin sensitivity. We hypothesized that with skin stretch, which occurs on the foot sole during dorsiflexion and the foot dorsum in plantarflexion, touch contact sensitivity and spatial acuity would decrease, whereas stretch sensitivity would increase. The opposite response is expected to occur when the skin is retracted. This is expected to occur at all sites on the foot sole and dorsum, excluding the heel where, previously, minimal change was observed in skin stretch or mechanics.

## Methods

### Subjects/Participants

Twenty healthy participants (11 female, mean ± SD: age 21.3 ± 1.4 years; mass 74.3 ± 11.5 kg; height 174.2 ± 9.0 cm) volunteered to participate in this study. The general public was recruited for this study through a departmental email and posters at the University of Guelph. The participants completed a health history questionnaire and were excluded if they had any neurological or musculoskeletal disorders, or any known allergies to adhesives. Each subject gave written informed consent prior to participating in the experiment. The protocol was approved by the University of Guelph’s Research Ethics Board.

### Protocol

**FIGURE 1:**
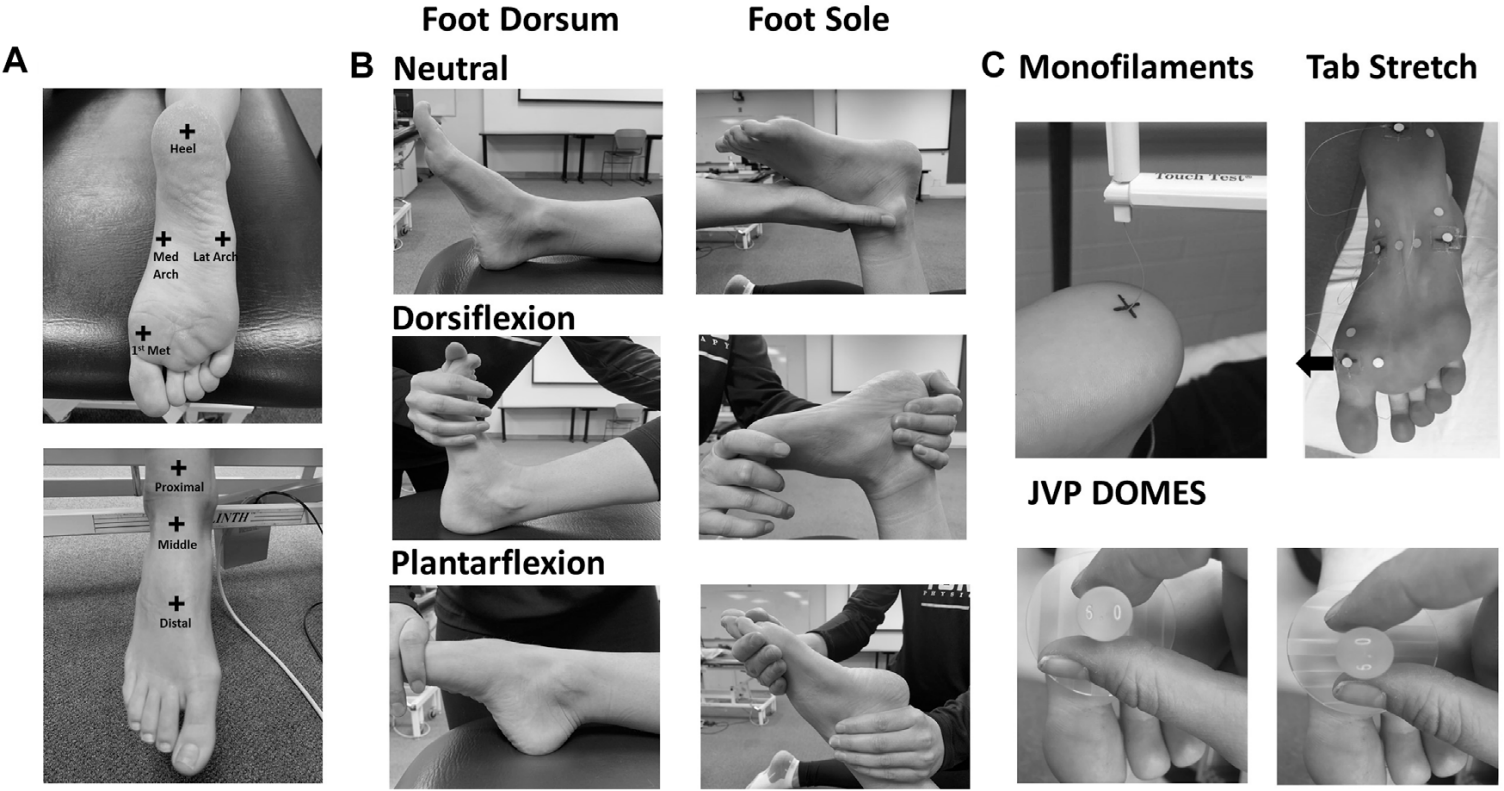
**(A)** Test sites on the foot sole and dorsum. **(B)** Foot positions in the neutral, dorsiflexion, and plantarflexion posture throughout testing on the foot sole and dorsum. **(C)** Skin touch sensitivity (monofilaments), stretch (tab stretch), and special acuity (JVP domes) sensitivity testing techniques.

The 20 participants were separated into two groups for testing on either the foot sole (*N* = 10, six female) or dorsum (*N* = 10, five female). There was no crossover of subjects between the two groups. Test sites were determined based on anatomical landmark proportions using the same protocol as Smith et al. (2019). Foot sole test sites included the heel, medial arch, and the first metatarsal (Figure 1A). The heel location was determined as the center of the heel at 15% of the foot sole length. The medial arch location was at 15% of the arch width from the medial border. The first metatarsal location was marked at 15% of the metatarsal width from the lateral and medial borders, respectively. For the foot dorsum, a region was marked with a proximal border at 15% of the length between the lateral malleoli and fibular head from the lateral malleoli, the distal border at 75% of the length between the lateral malleoli and fifth metatarsal from the lateral malleoli. The dorsum test sites included a proximal, middle, and distal site marked at 10, 50, and 90% from the proximal border of the reference region, respectively (Figure 1A).

Spatial acuity testing was also conducted on the foot sole lateral arch (in addition to the other three sites), which was measured as 15% of the arch width from the lateral border (Figure 1A). All 20 participants took part in the first day of collection. Touch and stretch sensitivity testing were conducted on the first collection day using Semmes-Weinstein monofilaments (Touch Test; North Coast Medical, Gilroy, CA) and a fastened skin tab, respectively (Strzalkowski et al., 2015b; Beaudette et al., 2017b). Eight foot sole participants and seven foot dorsum participants took part in an additional laboratory visit. A custom set of JVP domes designed for the foot and modeled after JVP^®^ domes (Stoelting Co., Wood Dale, IL, United States) were used for threshold and accuracy testing on the second collection day to determine spatial acuity. Each visit was approximately 2 hours in duration.

Throughout the protocol, the right foot was passively positioned into maximal dorsiflexion with toe extension, plantarflexion with toe flexion, and a neutral foot posture (Figure 1B). Maximum dorsiflexion and plantarflexion were defined as the maximal range of motion the participant was passively positioned into without discomfort. The subject’s foot was stabilized by an experimenter to prevent foot displacement during testing. To maintain motion-capture camera visualization, the subjects were lying supine with the right knee bent at approximately 45° and supported in the dorsiflexion and neutral posture during assessment of the foot dorsum. In the plantarflexion posture, the leg was placed in a straight position. The knee was bent at 90° with the subject lying prone for all postures during foot sole testing. Throughout testing the subjects were asked to relax all muscles to mitigate any effects of active muscle on skin deformation and perceptual tactile sensitivity measures. The same experimenter administered all sensitivity testing for all participants. The sensitivity tests (touch, spatial acuity, and stretch) were conducted for each participant, on either their foot sole or dorsum, but not both. The order of postures was randomized; however, the order of tests was maintained throughout all data collections. Skin stretch testing was conducted after monofilament touch testing to avoid the influence of adhesives on the tactile sensitivity assessment. Temperature at each test site was monitored throughout the protocol and humidity was consistent across participants and laboratory visits.

#### Touch Sensitivity

Skin touch sensitivity was assessed using Semmes-Weinstein monofilaments (Figure 1C). The monofilaments were applied normal to the skin of the test sites until the monofilament buckled. The monofilament was removed after approximately 1 second of application. This test was used to determine touch sensitivity threshold, which was defined as the smallest normal force that the subject could perceive with a 66% success rate, at all test sites in plantarflexion/toe flexion, dorsiflexion/toe extension, and the neutral postures. The subject’s sensitivity threshold was found using a modified 4-2-1 stepping algorithm (Dyck et al., 1993; Beaudette et al., 2017b). Following each monofilament application, subjects had to state whether the stimuli were perceived. To reduce anticipation bias, catch trials were randomly performed in which the investigator behaved in a manner similar to that used in the test trials; however, no monofilament was applied.

#### Spatial Acuity

Spatial acuity was evaluated using a two-interval forced choice (2IFC) grating orientation task performed with custom domes for use on the foot (Figure 1C). During each trial, the test site was contacted twice, with the same dome using orthogonal orientations (longitudinal and transverse axis of the foot) with random presentation. The participant was asked to indicate during which application the dome was applied with grooves along the transverse axis.

#### Dome Threshold

Threshold was determined in the neutral posture, with threshold defined as the groove width resulting in a correct orientation identification with 76% probability, corresponding to d-prime = 1 on this 2-IFC task (Gescheider, 1997). A modified adaptive Bayesian procedure (Peters et al., 2009) was used to determine threshold. During this test, domes (50 mm in diameter) with groove widths ranging from 1.0 to 30 mm were applied to the sites. Forty trials were conducted at each test site in the neutral posture to determine threshold.

#### Grating Orientation Accuracy

The dome corresponding to the threshold determined in the neutral ankle posture was subsequently applied to the test site for 20 applications in all three postures (neutral, dorsiflexion/toe extension, and plantarflexion/toe flexion) with a random presentation of dome orientation. As the subject could perceive this dome size with 76% probability, comparing the same dome in the various postures enabled us to assess any change in trial percent accuracy due to changes in sensitivity. The three postures were randomized to mitigate any learning effect. Percent accuracy (correct trials/total trials*100) at each site in the three postures was calculated and compared between postures.

#### Stretch Sensitivity

**FIGURE 2:**
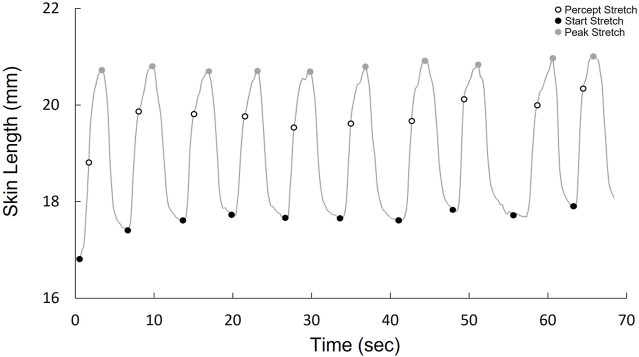
Kinematic stretch data over one trial (10 cycles), demonstrating the cycle onset, when stretch is perceived, and peak stretch for each cycle.

Stretch sensitivity was quantified using a method previously conducted on the trunk dorsum (Beaudette et al., 2017b). Small plastic tabs were affixed to the skin at each test site using a cyanoacrylate adhesive. Nylon cord was fastened along the longitudinal or transverse axis to allow for manual loads to be applied distally and medially on the tab. Reflective kinematic markers were adhered 20 mm proximal and lateral to the test site, as well as on the plastic tabs (Figure 1C). The displacement between the site marker and the proximal or lateral reference marker was used as a measure of local stretch along the longitudinal and transverse axis respectively. Marker position was recorded using a passive infrared optical system (Optitrack, Natural Point, Inc., Corvallis, OR, United States). The nylon cord was slowly pulled by the experimenter at a relatively constant velocity until the subject indicated that they perceived the sensation of skin stretch. A kinematic marker was placed on the subject’s right middle finger which the subject would flex when the sensation of skin stretch was perceived. The tension on the cord was then slowly released until no tension remained. Each tactile stretch sensitivity trial included 10 repeated loading cycles administered at random time intervals between 1–5 s (Figure 2). Longitudinal and transverse stretch ratios (SRs) were computed in alignment with the axis of the imposed stretch. The onset of acceleration of the finger marker was used to determine the skin displacement required for the subject to perceive stretch. The perceptual threshold was calculated as the mean across all 10 cycles. Both the longitudinal and transverse stretch were assessed at all sites in the three postures.

### Data Processing

#### Stretch Ratio

Raw kinematic data, sampled at 120 Hz, were filtered (4th order, dual-pass Butterworth) with a low-pass cut-off at 2 Hz. A three-point, finite central difference method was used to quantify the accelerations of the trigger marker through double-differentiated filtered 3D displacement data. To quantify each longitudinal or transverse SR, the 3D length (Euclidean norm) connecting either the longitudinal or transverse points was taken. Cycle-by-cycle SR values for each posture/location were calculated relative to the resting distance of each marker pair prior to each imposed loading cycle according to the following formula:

$$SR=\;D_{percept}/D_{original}$$

where SR is the stretch ratio at the perception of stretch and *D*_*percept*_ is the 3D distance between the markers calculated at each participant’s point of skin stretch perception for each imposed stretching load. *D*_*original*_ is the original 3D distance between the markers prior to each imposed stretching load.

### Statistical Analyses

IBM SPSS Statistics software was used to conduct statistical analyses. Data were assessed for normality using the Shapiro-Wilk test. All data were normal with the exception of the Semmes-Weinstein monofilament touch contact thresholds (mg) data. Friedman’s tests were conducted on the nonnormal data to assess for effects of Site and Posture. Pairwise comparisons were completed using Wilcoxon Signed Ranks test. Kendall’s W is given as a measure of effect size. Interactions between site and posture were not present, as determined by statistical graphics. General linear model, two-way repeated measures ANOVAs (Site) x (Posture) were conducted on the normally distributed data. Dependent variables included dome accuracy (%) and mean longitudinal and transverse stretch ratios (SR). Independent variables included the three ankle postures and the six test sites with an additional site for spatial acuity testing on the foot sole. A general linear model, one-way repeated measures ANOVA (for site differences) was applied to the dome threshold data set as it was only performed in the neutral posture. Post hoc comparisons were completed using a Tukey adjustment to identify the significant main effects or main effect interactions. Partial Eta Squared is given as a measure of effect size. All data are presented as means ± standard deviation (SD). A significance level alpha was set to 0.05 for all data. Descriptive statistics and graphs are reported as the means ± SD.

## Results

### Touch Sensitivity

#### Foot Sole

**FIGURE 3:**
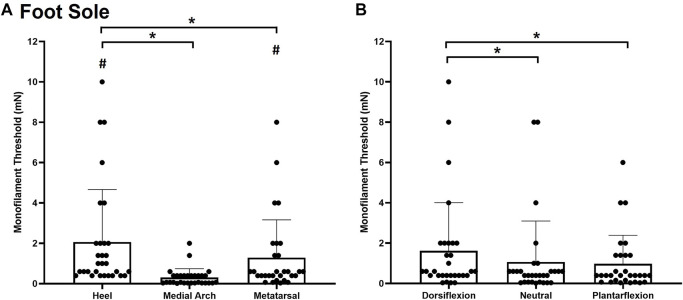
Mean (+SD) touch monofilament threshold at the test sites on the foot sole **(A)** and the three ankle postures **(B)**. Asterisks indicate a significant difference (*p* < 0.05). Pound symbols indicate a significant difference from the medial arch site **(A)** (*p* < 0.05). Dots represent threshold values for each participant.

Significant main effects of site χ^2^(2) = 43.000, *p* < 0.001, (Figure 3A) and posture χ^2^(2) = 11.186, *p* = 0.004 (Figure 3B), were observed for monofilament threshold. Specifically, the medial arch was significantly more sensitive to touch (0.31 ± 0.43 mg) compared to the heel (2.07 ± 2.60 mg, *p* < 0.001) and the metatarsal (1.29 ± 1.87 mg, *p* < 0.001). The metatarsal site required significantly less force to elicit perception compared to the heel (*p* = 0.004). Participants were also significantly less sensitive in the dorsiflexion posture (1.62 ± 2.39 mg) compared to the plantarflexion (0.98 ± 1.40 mg, *p* = 0.012) and neutral (1.07 ± 2.03 mg, *p* = 0.004) postures.

#### Foot Dorsum

**FIGURE 4:**
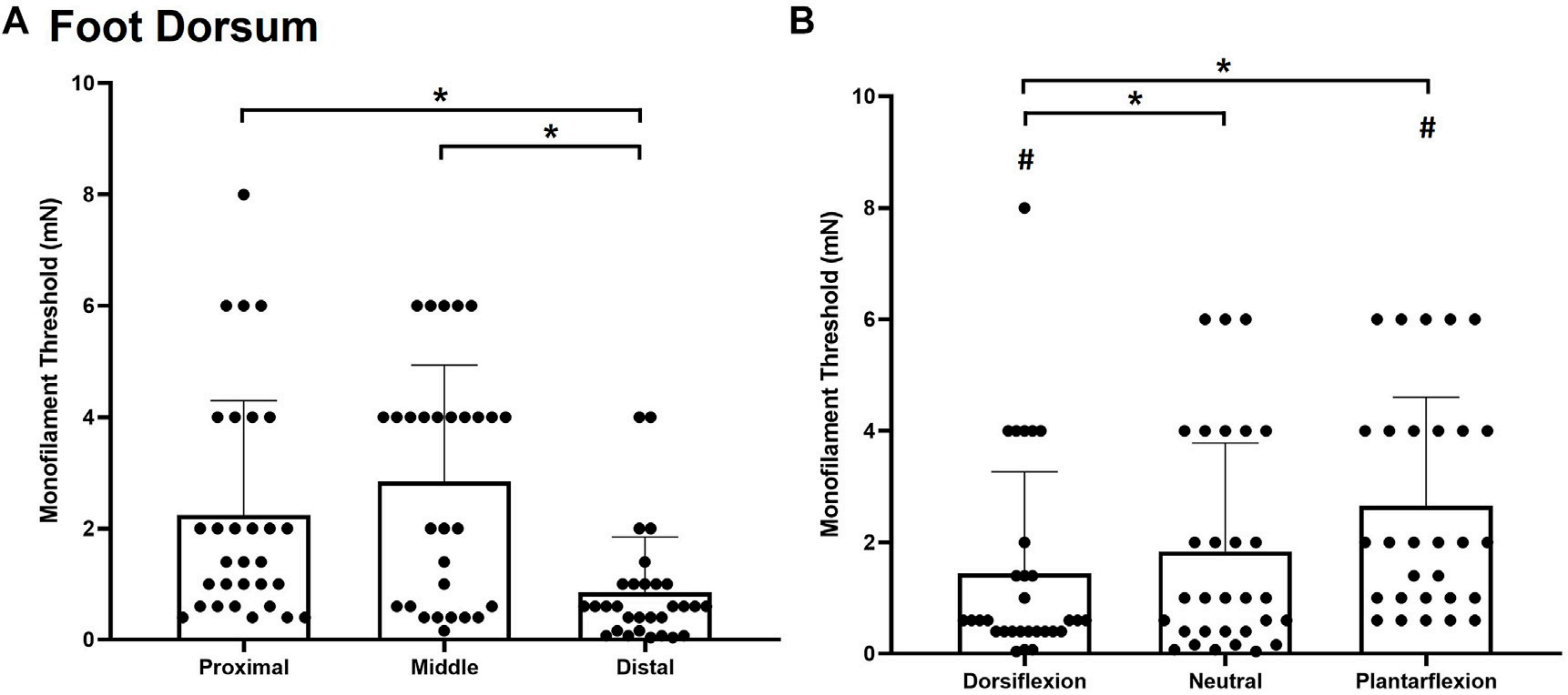
Mean (+SD) touch monofilament threshold at the test sites on the foot dorsum **(A)** and ankle joint postures **(B)**. Asterisks indicate a significant difference (*p* < 0.05). Pound symbols indicate a significant difference from the neutral posture **(B)** (*p* < 0.05). Dots represent threshold values for each participant.

Significant main effects of site χ^2^(2) = 27.185, *p* < 0.001, (Figure 4A) and posture χ^2^(2) = 28.059, *p* < 0.001, (Figure 4B) were observed for monofilament threshold. The distal site was the most sensitive to touch (0.8493 ± 0.9985 mg) followed by the proximal site (2.240 ± 2.055 mg; *p* < 0.001) and the middle site (2.84 ± 2.096 mg; *p* < 0.001). Subjects were also significantly less sensitive to touch contact in the plantarflexion posture (2.6483 ± 1.9579 mg) compared to the neutral (1.835 ± 1.945 mg, *p* < 0.001) and dorsiflexion ankle posture (1.4393 ± 1.8261 mg; *p* < 0.001). The dorsiflexion posture was significantly more sensitive than the neutral posture (*p* = 0.032).

### Spatial Acuity

#### Foot Sole

**FIGURE 5:**
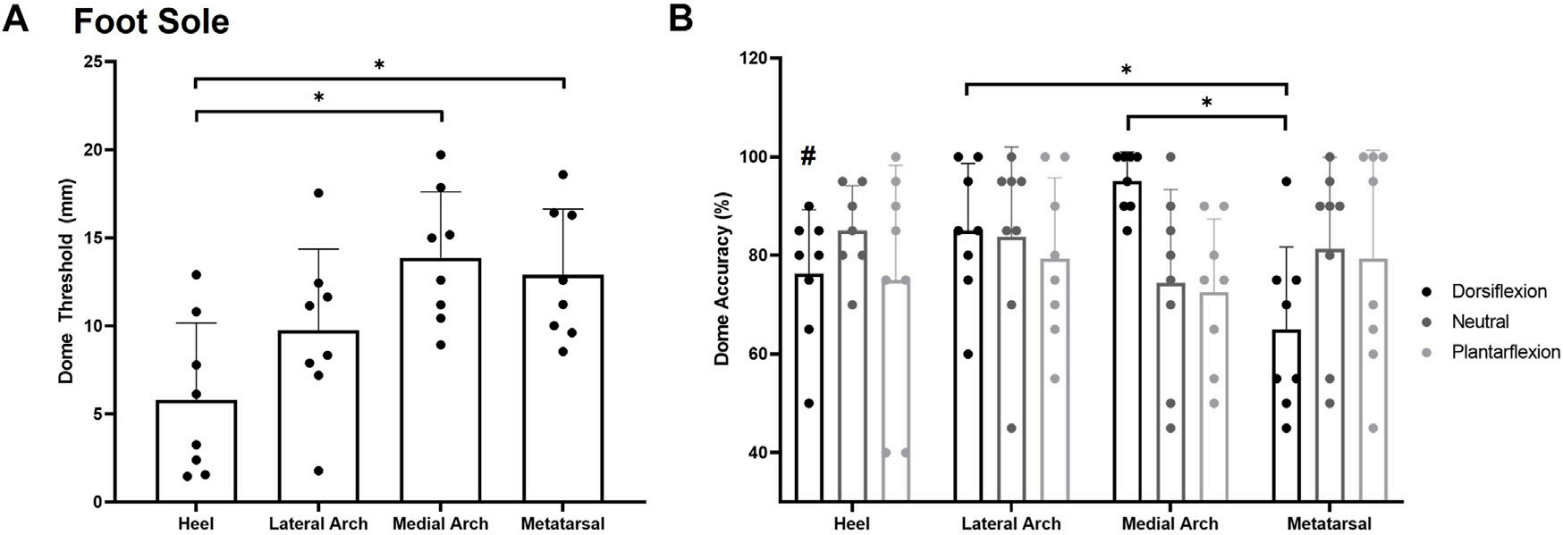
Mean (+SD) spatial acuity threshold across each of the foot sole testing locations **(A)**. Asterisks indicate statistically significant differences compared to the heel test site (*p* < 0.05). Mean (+SD) dome detection accuracy across each of the foot sole testing locations in the various postures **(B)**. Asterisks indicate a significant difference compared to the metatarsal site in the dorsiflexion posture (*p* < 0.05). Pound symbol indicates a significant difference compared to the medial arch in the dorsiflexion posture (*p* < 0.05). Dots represent threshold values for each participant. Note Figure A is in mm, where lower numbers signify greater sensitivity. Figure 5B is in % accuracy, where higher values indicate greater sensitivity.

For dome thresholds, a significant main effect of site *F*_(3,7)_ = 6.217, *p* = 0.002, η^2^ = 0.400 (Figure 5A) was observed on the foot sole in the neutral position. The heel (5.8 ± 4.4 mm) was significantly more sensitive than the medial arch (13.9 ± 3.7 mm; *p* = 0.003) and the first metatarsal (12.9 ± 3.7 mm; *p* = 0.011). The lateral arch was the second most sensitive test site with a threshold of 9.7407 ± 4.6150 mm.

For grating orientation % accuracy, a significant posture*site interaction was found *F*_(4,6)_ = 3.618, *p* = 0.006, η2 = 0.341. Participants were significantly more sensitive at the medial arch (95 ± 6%) compared to the heel (76 ± 13%; *p* = 0.027) and metatarsal (65 ± 17%; *p* = 0.001) sites in the dorsiflexion posture. The lateral arch (85 ± 14%) site was also significantly more sensitive than the metatarsal site in the dorsiflexion posture (*p* = 0.019). No significant main effect of site *F*_(3,7)_ = 0.528, *p* = 0.668, η2 = 0.070 or posture *F*_(2,7)_ = 0.718, *p* = 0.505, η2 = 0.093 was observed for grating orientation % accuracy on the foot sole (Figure 5B).

### Foot Dorsum

**FIGURE 6:**
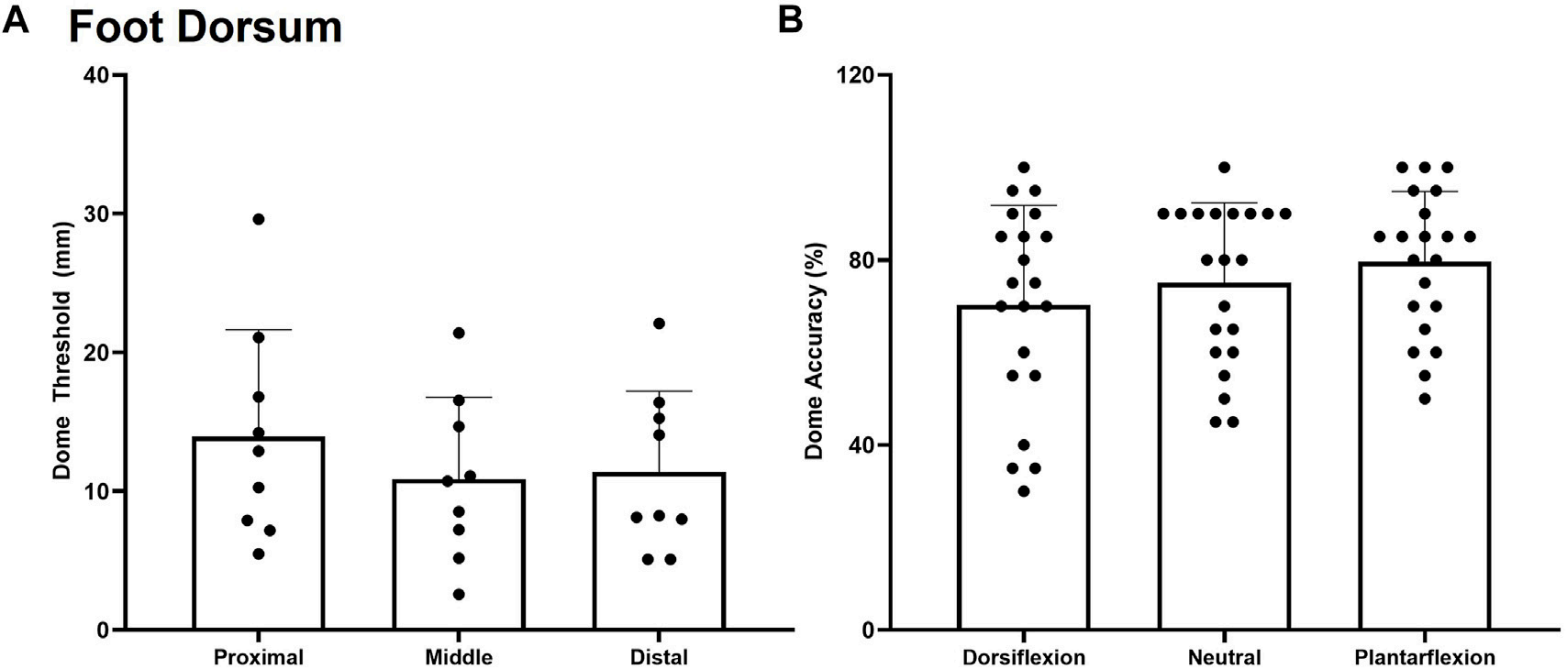
Mean (+SD) spatial acuity threshold across each of the foot dorsum testing locations. No significant difference was found between test sites **(A)** or trial accuracy on the foot dorsum for the various postures **(B)** (*p* < 0.05). Dots represent threshold values for each participant.

No significant effect of site *F*_(3,7)_ = 0.415, *p* = 0.666, η2 = 0.044 was observed for dome threshold on the foot dorsum in the neutral posture. The dome threshold for the proximal, middle, and distal test sites were found to be 12.9 ± 5.0 mm, 10.1 ± 6.3 mm, and 11.5 ± 5.9 mm respectively (Figure 6A). No significant effect of site *F*_(3,7)_ = 1.175, *p* = 0.342, η2 = 0.225, posture *F*_(2,7)_ = 1.743, *p* = 0.216, η2 = 0.164, or posture*site interaction *F*_(3,7)_ = 1.383, *p* = 0.270, η^2^ = 0.187 were observed for dome grating orientation % accuracy on the foot dorsum (Figure 6B).

## Stretch Sensitivity

### Foot Sole

**FIGURE 7:**
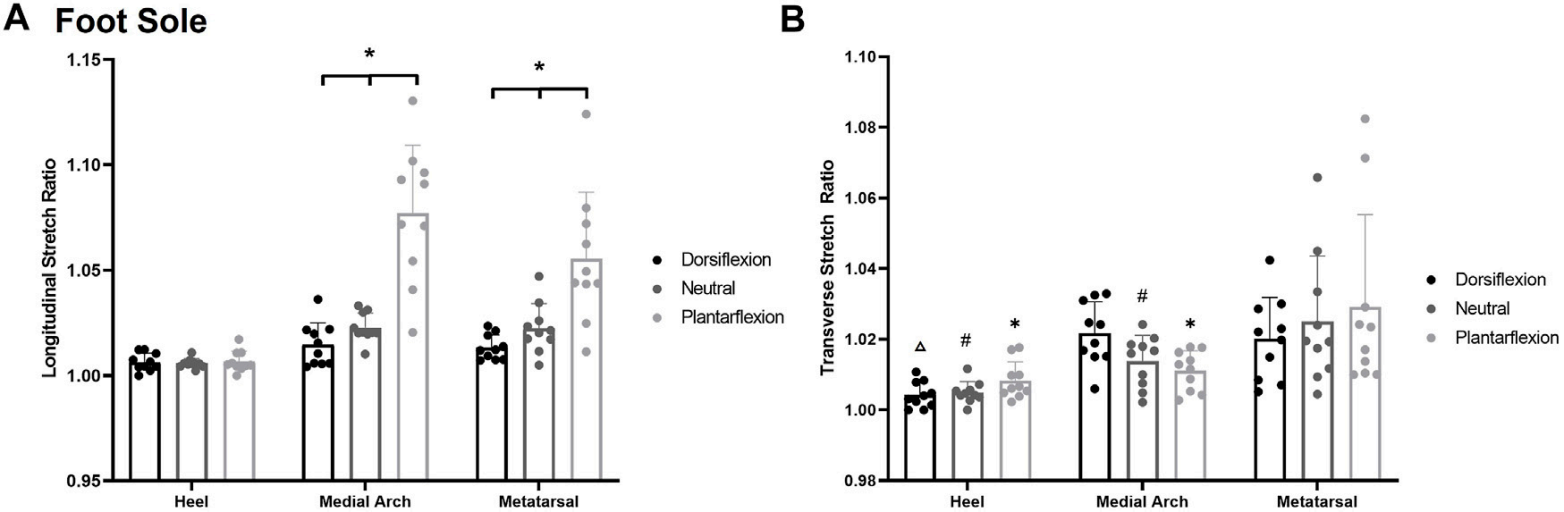
**(A)** Mean (+SD) longitudinal skin stretch threshold, expressed as a stretch ratio, across each of the foot sole testing locations for each posture. Asterisks indicate statistically significant differences between the various postures at the medial arch and metatarsal (*p* < 0.05). **(B)** Mean (+SD) transverse skin stretch threshold, expressed as a stretch ratio, across each of the foot sole testing locations for each posture. Asterisks indicate statistically significant difference from the metatarsal site in plantarflexion. Pound symbol indicates statistically significant difference from the metatarsal site in neutral. Triangle indicates statistically significant difference from the metatarsal site in dorsiflexion (*p* < 0.05). Dots represent threshold values for each participant. Note: an increase in stretch ratio indicates more stretch is required before the stretch is perceived.

A significant posture*site interaction was found for stretch sensitivity along the longitudinal axis *F*_(4,7)_ = 14.758, *p* < 0.001, η2 = 0.621 (Figure 7A). Subjects became less sensitive to skin stretch in the plantarflexion posture compared to neutral and dorsiflexion at all sites other than the heel (*p* < 0.001). Increased stretch sensitivity was also present in the heel compared to the first metatarsal and medial arch, demonstrated by stretch ratios of 1.006 ± 0.004, 1.030 ± 0.027, and 1.038 ± 0.034 respectively (*p* < 0.001). An increased stretch ratio indicates a greater change in stretch was required in the arch and metatarsal region before stretch was perceived.

A significant posture*site interaction was found for transverse stretch sensitivity *F*_(4,8)_ = 4.812, *p* = 0.003, η2 = 0.313 Figure 7B. The heel (1.009 ± 0.007) and the medial arch (1.011 ± 0.007) were significantly more sensitive than the metatarsal site (1.028 ± 0.026) in the plantarflexion (*p* < 0.001, *p* = 0.002) and the neutral posture (*p* < 0.001, *p* = 0.046). In the dorsiflexion posture, and the heel (1.003 ± 0.005) was significantly more sensitive than the medial arch (1.022 ± 0.006; *p* = 0.002) and the metatarsal (1.021 ± 0.010; *p* = 0.005). The medial arch and metatarsal sites were not significantly different from each other in the dorsiflexion posture (*p* = 0.779). Of note, there were no significant differences at each site across the various foot postures.

### Foot Dorsum

**FIGURE 8:**
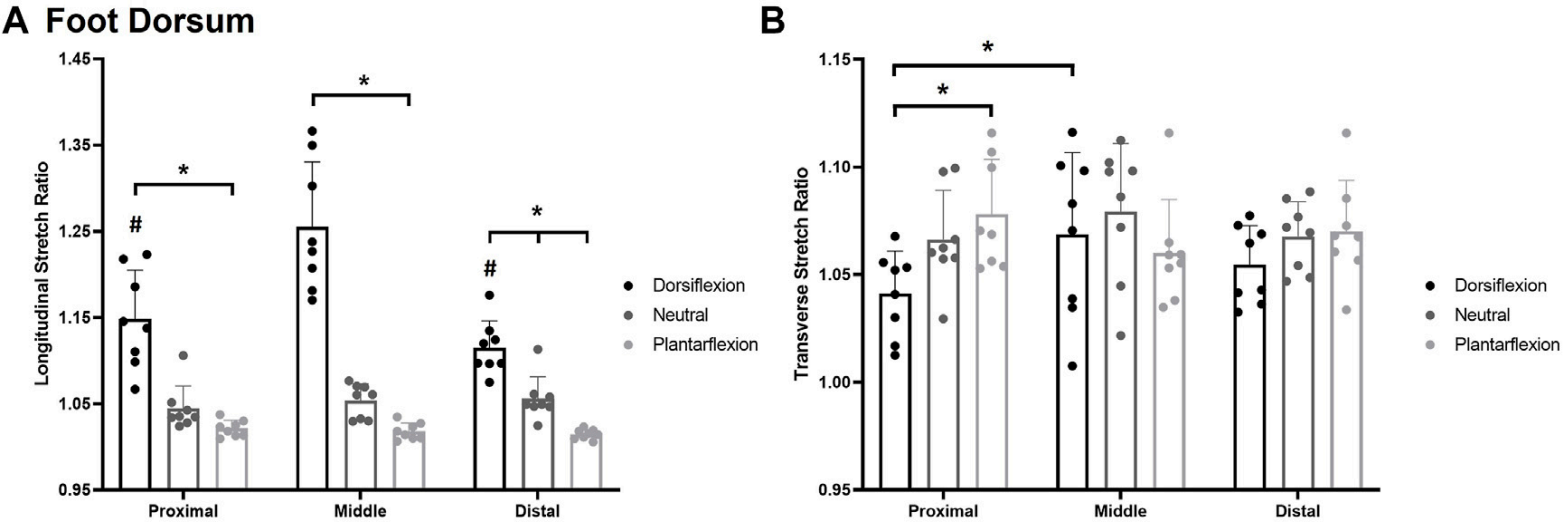
Mean (+SD) longitudinal skin stretch threshold, expressed as a stretch ratio, across each of the foot dorsum testing locations for each posture **(A)**. Asterisks indicate statistically significant differences between the various postures (*p* < 0.05). Pound symbol indicates statistically significant difference from the middle site in the dorsiflexion posture. Mean (+SD) transverse skin stretch threshold, expressed as a stretch ratio, across each of the foot sole testing locations for each posture **(B)**. Asterisks indicate statistically significant difference from the proximal site in the dorsiflexion posture. Dots represent threshold values for each participant. Note: an increase in stretch ratio indicates more stretch is required before the stretch is perceived.

A significant posture*site interaction was also observed for longitudinal stretch sensitivity on the foot dorsum *F*_(4,8)_ = 17.628, *p* < 0.0014, η2 = 0.716 (Figure 8A). Participants were significantly more sensitive in the plantarflexion posture (*p* < 0.05) at each test site compared to the dorsiflexion posture. The distal site was also significantly more sensitive in the plantarflexion posture (1.015 ± 0.005) compared to the neutral posture (1.056 ± 0.025; *p* = 0.0025) whereas the proximal site (*p* = 0.205) and middle site (*p* = 0.053) were not significantly different between plantarflexion and neutral. In the dorsiflexion posture, the middle test site (1.256 ± 0.076) was also significantly less sensitive than the proximal (1.150 ± 0.056; *p* < 0.001) and the distal (1.115 ± 0.032; *p* < 0.001) test sites.

A significant posture*site interaction was also found for transverse stretch *F*_(4,8)_ = 5.048, *p* < 0.003, η2 = 0.419 Figure 8B. In the dorsiflexion posture, the proximal site (1.041 ± 0.020) was significantly more sensitive than the middle site (1.069 ± 0.039; *p* = 0.033). The proximal site was also significantly more sensitive in dorsiflexion (1.041 ± 0.020) compared to the plantarflexion posture (1.079 ± 0.027; *p* = 0.005).

## Discussion

The purpose of this study was to assess the effect of foot posture on perceptual sensitivity of the foot sole and dorsum. In addition to corroborating previous accounts of location dependent sensitivity, we found that foot posture influences tactile perceptual sensitivity measures. Measures of stretch and monofilament tactile input, in particular, were influenced by posture. On the plantar surface, stretch sensitivity, which primarily activates SAII receptors, was found to increase during dorsiflexion and decrease during plantarflexion at all locations except the heel. On the foot dorsum, stretch sensitivity increased with plantarflexion and decreased with dorsiflexion. Touch sensitivity, which is believed to target FAI receptors, was reduced in plantarflexion and increased in dorsiflexion on the foot dorsum. On the foot sole, touch sensitivity increased in plantarflexion and was reduced in dorsiflexion. Finally, spatial acuity increased at the medial arch in the dorsiflexion posture but decreased at the metatarsal in the same posture. The results suggest differing effects of ankle posture on the various skin receptors, which may provide insight into target regions for intervention during dynamic tasks.

### Monofilament Touch Sensitivity

#### Location

Touch sensitivity thresholds in the neutral ankle joint posture were similar to previous reports that assessed sensitivity on the foot sole (Strzalkowski et al., 2015a) and dorsum (Mildren et al., 2017). Monofilaments are known to activate FAI afferents at the onset of perception (Strzalkowski et al., 2015a). As such, perceptual thresholds using monofilaments are an ideal measure of how foot position affects FA afferents. In the current work we found that the heel was the least sensitive and the medial arch had the greatest sensitivity, which follow data from previous accounts (Strzalkowski et al., 2015a; Strzalkowski et al., 2015b). The location differences in sensitivity on the foot sole were once thought to relate to the density of receptors in the region of interest (Kennedy and Inglis, 2002). Recent density calculations in the foot sole refute this, with data instead supporting the medical arch as the most sensitive region of the foot sole despite its lowest density of receptors (Strzalkowski et al., 2018). Other work has looked to different contributors to explain the location-based sensitivity across the foot sole such as location differences in mechanics. Strzalkowski and colleagues (2015a) have shown that the location dependent mechanical properties of the skin on the foot mirror the location dependency of threshold sensitivity, as well as firing onset of FAI afferents (Strzalkowski et al., 2015a). The skin of the medial arch is the softest region on the foot sole, while the heel region is the hardest (Strzalkowski et al., 2015b; Smith et al., 2019), supporting the idea that mechanics, rather than density, affect perception of touch activating FAI afferents. A location dependent response was also demonstrated on the foot dorsum. Similar to the medial arch on the foot sole, the distal test site is the most sensitive and the thinnest region on the foot dorsum (Smith et al., 2019). Previous work on the foot dorsum, with sites comparable to the present study, demonstrated increased sensitivity on the distal sites compared to the proximal ones (Hennig and Sterzing, 2009; Mildren et al., 2017). Differences in local mechanics may therefore contribute to changes in perceptual sensitivity of FAI receptors across the foot.

#### Posture

On the foot sole, individuals were significantly more sensitive in the plantarflexion posture than dorsiflexion. However, on the foot dorsum, subjects were significantly more sensitive in dorsiflexion compared to plantarflexion. Greater sensitivity was therefore present when the skin was compressed rather than stretched. Skin stretch due to ankle posture correlates with increased skin hardness, whereas skin compression is associated with softer skin (Smith et al., 2019). As previously mentioned, regions on the foot sole with greater skin hardness are less sensitive than softer regions (Strzalkowski et al., 2015a). Changes in hardness due to skin stretch may therefore be in part responsible for changes in cutaneous sensitivity as these mechanical properties can impact deformation in response to indentation (Takei et al., 2004; Staloff and Rafailovitch, 2008), altering the ability to transmit forces and activate cutaneous mechanoreceptors.

### Spatial Acuity

#### Location

The grating orientation task is a validated, objective test for spatial acuity which has been previously conducted on the hand (Craig, 1999; Liboutona et al., 2012; Tremblay et al., 2000). However, grating orientation thresholds across the foot are unknown. Customized domes were designed for the foot to measure spatial acuity, which is partially influenced by receptor density of the SAI afferent ending (Johnson and Hsiao, 1992). Our adapted domes aimed to capture differences in spatial acuity across the foot sole and dorsum skin. Our results showed that the heel was the most sensitive test site, followed by the lateral arch. The medial arch and the first metatarsal were the least sensitive regions with similar thresholds. To provide insight for the region-specific sensitivity, we can look to microneurography studies to find supporting data based on calculations of receptor density. Recent data suggest increased mechanoreceptor density in a gradient toward the toes and the lateral region of the foot sole (Strzalkowski et al., 2018). Interestingly, these recent reports on receptor densities do not correlate with the grating orientation thresholds found in the present study, whereby the most sensitive region was found to be the heel, a location that reportedly has fewest receptors, with foot sole density increasing in a gradient towards the toes (Strzalkowski et al., 2018). Factors other than receptor population must therefore contribute to skin spatial acuity. Increased sensitivity across the heel may in part be due to greater weighting of this skin region in the primary somatosensory cortex (Johansson and Vallbo, 1979; Craig, 1999). It has been previously shown that hand regions which are more functionally significant are more highly represented in the central nervous system, resulting in increased sensitivity (Johansson and Vallbo, 1979; Craig, 1999). A similar mechanism may be present on the foot sole, contributing to greater spatial acuity across the heel and lateral aspect of the foot. If we argue that pressure resolution, as signaled by SAI (Liu et al., 2020), is critical for contact during gait and other functions of the foot, lower mechanoreceptor density at the heel must be offset by increased central importance.

#### Posture

In the current study, the medial arch was significantly more sensitive in the dorsiflexion posture than the heel and the metatarsal sites. In the dorsiflexion posture, the plantar fascia is stretched, resulting in a taught ligament under the testing region on the medial arch (Aquino and Payne, 1999). This additional cue may improve spatial acuity at the medial arch in the dorsiflexion posture. In contrast, the metatarsal site was significantly less sensitive than the lateral arch in the dorsiflexion posture. We believe this is due to a limitation in the study design. In the dorsiflexion posture, less of the dome surface is in contact with the metatarsal region due to toe extension, decreasing the tactile information available and likely resulting in the decreased accuracy demonstrated in this study.

Overall, percent accuracy on the grating orientation task did not change in the various postures across foot sole or dorsum in the current work. Based on these findings, grating orientation thresholds, it seems, were not influenced by mechanics, which is in contrast to FAI thresholds/touch sensitivity investigated via Semmes-Weinstein monofilaments. These responses to mechanical differences may be largely due to the method of testing whereby the domes were applied above the low threshold levels for touch perception and as such are likely not as impacted by skin mechanics across location.

Spatial acuity testing on the trunk (Beaudette et al., 2017b) and wrist (Cody et al., 2010) have reported sensitivity is reduced in postures that stretch the skin. The insignificant postural changes in the present study may be due to the limited range of motion of the ankle joint resulting in less skin deformation compared to the trunk dorsum and wrist. Normalized values of extension/flexion range of the ankle, trunk, and wrist are 70°, 110°, and 150° respectively (Clarkson, 2000). It was hypothesized that as skin stretched over the ankle joint, the SAI receptors would move apart from each other, lowering the density of the receptors and therefore spatial acuity. Ankle joint position may not result in large enough skin deformation to affect spatial acuity of the foot sole and dorsum.

### Stretch Sensitivity Threshold

#### Location

Stretch sensitivity has been previously studied on the hand (Edin 1992; Edin, 2004), the back (Beaudette et al., 2017b), and recently the thigh (Macdonald et al., 2019). Foot sole skin has the unique characteristic of weight bearing and withstanding large external stresses (Swensson et al., 1998). As a result, the structure of the plantar surface is unique (Swensson et al., 1998). The skin of the heel is the least elastic region on the foot sole, resulting in the least amount of stretch and deformation (Dohi et al., 2004; Parker 2013). This is further demonstrated by significant skin stretch occurring at all tested sites on the foot sole other than the heel when participants move from maximal dorsiflexion to plantarflexion (Smith et al., 2019). In the current study, the heel site was more sensitive to longitudinal and transverse stretch than the other sites on the foot sole, likely due to the heel skin’s limited ability to deform. The skin of the heel is unable to stretch as far as other regions, resulting in lower stretch ratios in comparison. This finding supports our previous work (Macdonald et al., 2019), where we propose that regions of skin with increased stretch properties have reduced sensitivity to this stretch in order to mute feedback within its normal functional range. Muting of input would correspond to amplification of skin feedback outside of this range.

The hairy skin of the foot dorsum has greater capability to stretch than skin of the foot sole due to structural differences between hairy and glabrous skin (Edin, 1992). Hairy skin lacks the tight connections to subcutaneous tissues present on glabrous skin, allowing for less resistance in response to joint movement (Edin, 1992). Skin on the foot dorsum is not subject to the large pressures on the foot sole which can result in regional changes to elasticity (Valle and Zamorani, 2007). As such, foot dorsum skin is more uniform in elasticity, resulting in similar stretch sensitivity over the test sites.

#### Posture

On the foot sole, participants were more sensitive (took the least amount of stretch for perceptual detection) in the dorsiflexion posture compared to plantarflexion along the longitudinal axis. Participants were therefore more sensitive when the skin was “pre-stretched.” It has previously been hypothesized that “pre-stretch” of the skin brings SAII receptors, the cutaneous mechanoreceptors believed to be responsible for the sensation of stretch, closer to activation threshold (Beaudette et al., 2017b). Less additional stretch is therefore required to evoke the sensation of stretch. The same response was shown with longitudinal stretch on the foot dorsum. Participants were more sensitive when the skin was “pre-stretched” during plantarflexion compared to dorsiflexion. Transverse stretch sensitivity was not significantly altered between the various postures. Greater posture related changes in sensitivity are expected along the longitudinal axis as it is along the same plane of movement as dorsiflexion/plantarflexion.

### Limitations

A limitation in the study design for spatial acuity testing included a lack of full contact between dome surface and metatarsal region in the dorsiflexion posture. We believe the decreased tactile information available likely resulted in a decreased percent accuracy found in this posture. As such, it is not possible to make significant conclusions around this data point and future work will need to explore the sensitivity of the SAI population in this region. One further notable limitation in the current work is the presence of calluses that may appear over the foot surface, as the increased skin hardness may affect touch sensitivity (Strzalkowski et al., 2015b). To mitigate the effects of calluses on touch sensitivity measures, if a callus was present over the monofilament test site, monofilament testing was conducted at the closest region where a callous was not present. Calluses were deemed to have little impact in stretch tests as the whole region of skin was translated, or in spatial acuity testing, where the area of the dome was significantly larger than any calluses that were encountered. Calluses were found rarely in our participant pool at the locations of interest. Lastly, a variety of sensitivity tests were chosen for this study. Each test targets a specific class of mechanoreceptors, allowing us to capture the effects of posture on the individual classes. The sensitivity changes captured in the various tests occur due to different structural changes in the skin, such as hardness, thickness, and receptor density. As such, it is unlikely the changes in the various measures would be related; however, correlations between the tests were not conducted in the current study.

### Implications

Understanding the processing of tactile information is crucial for the development of interventions that target cutaneous mechanoreceptors. Functionally, it is important to assess how sensory information is altered due to ankle posture and distribution of pressure across the foot sole. The ankle joint undergoes a range of postures throughout the gait cycle. Depending on the class of cutaneous mechanoreceptors targeted, skin interventions around the ankle and foot may need to consider skin stretch and retraction patterns that could occur during different points in the gait cycle. This would help to evaluate appropriate locations for vibration, taping, or fabrics to help enhance skin feedback. Additionally, a recent study (Lung et al., 2020) demonstrated that plantar tissue stiffness increases with increased walking speeds. Based on our results, this would likely modify skin sensitivity. Additional research is necessary to determine changes in cutaneous sensitivity during functional tasks and to optimize the location of biofeedback interventions.

## Conclusion

Based on our current data set we conclude that tactile perceptual sensitivity on the foot differs according to ankle joint posture. Sensitivity to touch contact and stretch were altered with ankle posture, whereas spatial acuity was not greatly impacted, except for one site in one posture. Changes in sensitivity across locations and postures were believed to occur due to concurrent changes in skin mechanics, such as skin hardness and thickness as shown across similar ankle postures in our recent paper (Smith et al., 2019). Future cutaneous biofeedback interventions should be applied with an awareness of these changes in skin sensitivity, to maximize their effectiveness for foot sole and dorsum input.

## Data Availability

The original contributions presented in the study are included in the article/Supplementary Material, further inquiries can be directed to the corresponding author.
